# Efficacy and Safety of TurmXTRA® 60N in Delayed Onset Muscle Soreness in Healthy, Recreationally Active Subjects: A Randomized, Double-Blind, Placebo-Controlled Trial

**DOI:** 10.1155/2022/9110414

**Published:** 2022-08-05

**Authors:** Shefali Thanawala, Rajat Shah, Vasu Karlapudi, Prabakaran Desomayanandam, Arun Bhuvanendran

**Affiliations:** ^1^Nutriventia, Inventia Healthcare Ltd., Mumbai, India; ^2^Pujitha Hospital, Vijayawada, India; ^3^In Vitro Research Solutions (iVRS) Pvt Ltd, Bengaluru, India

## Abstract

**Background:**

Delayed onset of muscle soreness (DOMS) and its physiological consequences influenced an individual's adherence to an exercise routine.

**Objective:**

This study aimed to evaluate the efficacy, safety, and tolerability of TurmXTRA® 60N (WDTE60N) on DOMS compared to placebo in recreationally active healthy subjects.

**Methods:**

This randomized, double-blind, placebo-controlled parallel-group study enrolled 30 healthy and recreationally active subjects (average age: 28.23 ± 4.20 years) and randomized them to receive WDTE60N (WDTE60N group; *n* = 15) or placebo (placebo group; *n* = 15). Study treatments were initiated 29 days before the eccentric exercise and were continued for 4 days after the exercise. The primary endpoint was the change in pain intensity measured by the visual analog scale (VAS) at the end of study treatment (at 96 hours after eccentric exercise) from baseline (measured immediately after exercise).

**Results:**

The VAS score indicated that subjects from the WDTE60N group reported significantly less pain after eccentric exercise compared to the placebo group (AUC_0–96h_: 286.8 ± 46.7 vs. 460 ± 40.5, respectively; *p* < 0.0001). Well-being status was assessed using the adapted version of the Hooper and MacKinnon questionnaire and was calculated as individual and cumulative scores of the domains (fatigue, mood, general muscle soreness, sleep quality, and stress) that demonstrated improvement in all domains and in overall well-being in the WDTE60N group compared to the placebo group (*p* < 0.0001). Serum lactate dehydrogenase (LDH) was significantly lower in the WDTE60N group compared to the placebo group (AUC_0–96h_: 23623.7 ± 2532.0 vs. 26138.6 ± 3669.5, respectively; *p*=0.0446).

**Conclusion:**

Intake of WDTE60N before and after eccentric exercise significantly reduced subjective perception of muscle soreness and serum LDH activity and increased the psychological well-being in recreationally active subjects.

## 1. Introduction

Delayed onset muscle soreness (DOMS) is a type of ultrastructural muscle damage, that is, caused by high-intensity eccentric muscle contractions or unaccustomed forms of exercise [1]. The symptoms include impaired muscular force capacities, muscle stiffness, aching pain, and/or muscular tenderness with some altered biomechanics to the adjacent joints [2–4]. It usually begins 6–12 hours after completing the exercise, peaks at 48–72 hours, and slowly resolves in 5–7 days [5]. However, it is one of the responsible elements for compromised sports performances. Thus, recovery is of utmost importance as it allows for a quicker return to the next bout of the exercise and improves adaptations and performance in subsequent exercise bouts. Several dietary supplements are valuable in enhancing muscular adaptations to exercise and help in improving brain performance, decreasing DOMS or pain, reducing injury severity, and enhancing recovery from injury. Notably, these dietary supplements do not produce ergogenic effects but assist the athletes to train and/or compete more effectively without performance impairments [6].

Curcumin is a natural polyphenolic substance that constitutes 77% of the total curcuminoids present in the turmeric rhizome. It gives a distinctive yellow color to the spice turmeric. Owing to its numerous physiological effects, since ancient times, it has been involved in traditional Indian and Chinese medicine. Evidence demonstrates its beneficial effects in treating cancer, cardiovascular health, arthritis, and Alzheimer's dementia indicates its anticarcinogenic, anti-inflammatory, antioxidant, and neuroprotective properties [7–10]. Curcumin produces analgesic effects on acute and chronic pain by desensitizing the transient receptor potential vanilloid 1, an ion channel responsible for pain sensation, thereby reducing pain sensitivity [11]. It also decreases postexercise inflammation by regulating nuclear factor kappa B (NF-*Ƙ*B) and nuclear factor erythroid 2-related factor 2 (Nrf2) pathways that ultimately reduce pain sensitivity and DOMS [12]. It is hypothesized that curcumin, being a free radical scavenger, reduces secondary muscle damage after exercise. Taking into consideration the proven benefits of curcumin, the International Olympic Committee consensus classified it as a nutritional supplement that might improve training capacity, recovery, muscle soreness, and injury management [13].

One of the factors that limit its widespread use is—its poor oral bioavailability, however, this can be overcome by administering very large doses (approximately 6–8 g) of turmeric powder or 1500–2000 mg of turmeric extract standardized to 95% for gaining optimal effects. However, long-term administration of such high doses of curcumin is discouraged as they can cause gastrointestinal side effects (intestinal disturbances and urticaria) and poor compliance [14]. TurmXTRA®60N (WDTE60N; of Nutriventia, Inventia Healthcare Ltd.), a novel formulation of the natural, water-dispersible turmeric extract containing 60% natural curcuminoids, is developed using patented technology to overcome the aforementioned limitations of curcumin. A preclinical pharmacokinetic study using Sprague Dawley rats demonstrated ten-fold higher bioavailability with WDTE60N compared to standard turmeric extract 95% [15]. These results have been reciprocated in a comparative pharmacokinetic study that evaluated the pharmacokinetics of WDTE60N compared to standard turmeric extract 95% in healthy subjects [16]. Findings of this study indicate that water dispersibility of WDTE60N aids in the absorption of active moieties resulting in higher levels of plasma-free curcumin, total curcumin, and total curcuminoids compared to standard turmeric extract of 95% when subjects were administered a ten-fold lower quantity of curcuminoids in WDTE60N. Recently, we assessed the efficacy of WDTE60N in alleviating symptoms of chronic knee pain [17]. Results of the randomized study confirmed significantly greater pain reduction among those receiving WDTE60N compared to those, who received placebo as was evident by a significant reduction in VAS score (−1.5 ± 0.7 vs −0.6 ± 0.8, *p* < 0.0001). Furthermore, the results for the time taken for 80 m fast-paced walk test and 9-step stair-climb test with WDTE60N intake were also satisfactory.

Thus, this randomized controlled trial was designed to assess the efficacy, safety, and tolerability of TurmXTRA® 60N (WDTE60N) compared to placebo on DOMS in recreationally active subjects. We hypothesized that consistent use of a single daily dose of 250 mg TurmXTRA® 60N during pre- and post-period of eccentric exercise reduces DOMS and muscle damage.

## 2. Materials and Methods

### 2.1. Study Design

The present randomized, double-blind, placebo-controlled parallel-group study was conducted between 29 Dec 2021 and 07 Feb 2022 after receiving approval from an institutional ethics committee of the study center (ACE Independent Ethics Committee, registration detail: ECR/141/Indt/KA/2013/RR-19). The study was conducted following the pertinent requirements of the Declaration of Helsinki (Brazil, October 2013), Good Clinical Practices for Clinical Research in India 2005, New Drugs and Clinical Trials Rules 2019, ICH E6 (R2), Guidance on Good Clinical Practice, and with ICMR's National Ethical Guidelines for Biomedical and Health Research Involving Human Participants-2017. All subjects were thoroughly informed about the study before their enrollment, and before obtaining their signed informed consent forms. This study was registered with the Clinical Trial Registry of India (registration no. CTRI/2021/12/038892). The study is reported in accordance with the CONSORT 2010 reporting guideline (Supplementary Table 1).

### 2.2. Study Population

The present study was conducted on 30 healthy and recreationally active individuals. The subjects were included if they met the following inclusion criteria: (1) Male and female subjects (ages 18–35 years) had a body mass index of 18–30 kg/m^2^; (2) Healthy, moderately active (regular aerobic exercise for at least 150 min per week for past 3 months), nonsmoker subjects without any known musculoskeletal pathology; (3) Subjects vaccinated (two doses) for Coronavirus disease 2019 (COVID-19); (4) Female subjects of childbearing potential were included only if they agreed to use a medically acceptable form of birth control; (5) Subjects willing to sign the informed consent and comply with the study procedure; (6) Subjects willing to abstain from using analgesics/nonsteroidal anti-inflammatory drugs during the treatment period; and (7) Subjects, whose screening test results were within the normal range or were not considered clinically significant by the principal investigator. However, the subjects were excluded from the study if female subjects were pregnant, breastfeeding, or were planning to get pregnant; subjects had COVID-19 symptoms; subject had known allergy to any compound present in the study product; subject was regularly involved in strength training; subjects had a history or frequently uses over-the-counter nonsteroidal anti-inflammatory drugs and/or analgesic agents; subjects with high alcohol intake (>2 standard drinks per day) or use recreational drugs (cocaine, methamphetamine, and marijuana), or have nicotine/caffeine dependence; subjects with a history/presence of chronic illness including but not limited to psychiatric (psychosis, major depressive disorder, or other clinically significant psychiatric disorder), cardiovascular, pulmonary, neurological, gastrointestinal, hepatic, renal, metabolic, and ophthalmologic disorders, or with human immunodeficiency virus infection and acquired immune deficiency syndrome, or those taking regular prescription pharmacological agents; subjects who participated in any other trials involving investigational or marketed products within 30 days prior to the screening visit; subjects who underwent surgery or trauma affecting the knee in the past; subjects with a history or presence of any neurological or muscular disorders that may affect muscle strength; and subjects regularly taking multivitamins/herbal and/or turmeric supplements/any other wellness product.

### 2.3. Study Procedure

The study was conducted at the Arogyavardhini Ayurvedic Center in Bangalore, Karnataka, India. All subjects were screened and evaluated 7 days before administering study treatments. Screening evaluation included anthropometry measurements, vital sign assessment, physical examination, medical and medication history (including a history of substance abuse and or addiction to drugs, cigarette smoking and alcohol intake, any concomitant medication usage), urine pregnancy test for female subjects with childbearing potential, electrocardiogram, laboratory investigations for liver function, renal function, hematology, fasting blood glucose, and human immunodeficiency virus infection. All subjects were given a COVID-19 screening questionnaire and those, who fulfilled all the eligibility criteria were randomly allocated to two treatment groups, the WDTE60N group (*n* = 15) or the placebo group (*n* = 15) using the block randomization on day 1. The randomization schedule was generated using SAS version 9.4 (SAS Institute Inc., USA) by an independent statistician and it was made available to the study personnel responsible for packaging, labeling, and blinding the investigational product. The subjects were enrolled by the study investigator. Blinding was performed via a computer-generated process. The study team statistician was responsible for the blinding process. The randomization numbers were preprinted as stickers and were affixed on every bottle, and this was the only way to identify one kit from the other. Investigator assigned treatment kits to the enrolled subjects as per the randomization schedule. The subjects were instructed to administer the study treatment (either a capsule containing 250 mg water-dispersible turmeric extract ((WDTE60N) or placebo) once daily, orally with adequate water after breakfast. The subjects were required to administer the study products for 33 days (a preexercise period of 29 days and a postexercise period of 4 days). They were also asked to maintain a subject diary that would include all the details regarding their daily intake of study products and concomitant medication (if any). Dosing compliance was assessed by reviewing each subject diary at every visit.

Subjects performed eccentric exercise (squatting) on Day 29. Squatting involves quadriceps muscles to undergo eccentric contractions in the downward phase of the movement. According to eccentric exercise protocol, each subject was required to perform one set of squats (measured as 15 squat cycles) in one minute and they performed this for 15 minutes to complete 225 squat cycles. However, they could stop the exercise if they were unable to continue (usually when the neuromuscular system can no longer produce adequate force to proceed) or when the investigator felt that the exercise was sufficient to induce DOMS.

### 2.4. Study Endpoints

The primary efficacy endpoint was an improvement in pain intensity from baseline (immediately after eccentric exercise) to the end of the treatment (at 96 hours after eccentric exercise).

The pain was assessed by asking the question, “what is the intensity of your current pain?” and the response was recorded on a visual analog scale (VAS) that had a 10 cm line with “no pain” on one end and “worst imaginable pain” on the other end. This was measured immediately before, immediately after (baseline), and at 12, 24, 48, 72, and 96 hours after eccentric exercise.

The secondary efficacy endpoints of the study included improvement in the domains of fatigue, sleep quality, general muscle soreness, stress level, mood, and overall well-being from baseline (immediately after exercise) to the end of treatment (at 96 hours after exercise) that were evaluated based on the adapted version of the Hooper and MacKinnon questionnaire. The adapted version was used for ease of administration in the study.

Self-reported ratings of well-being may be efficient in monitoring overtraining and recovery. The Hooper and MacKinnon questionnaire quantified and qualified the well-being relative to fatigue, sleep quality, general muscle soreness, stress, and mood [18]. This questionnaire is often used as a repeated measure to investigate the changes over time in individuals or as a general indicator of well-being across the group to understand general patterns [19]. Furthermore, it is found sensitive to variations within and between weeks [20]. This questionnaire was later updated by McLean et al. in 2014 [21]. The adapted version of the Hooper and MacKinnon questionnaire provides a subjective assessment of the impact of DOMS in the domains of fatigue, sleep quality, general muscle soreness, stress level, and mood on a scale of 1–5, with higher scores denoting the better results.

In the present study, the well-being of subjects was assessed using the adapted version of the Hooper and MacKinnon questionnaire immediately before, immediately after (baseline), and at 12, 24, 48, 72, and 96 hours after eccentric exercise.

Changes in serum creatine kinase (CK) and lactate dehydrogenase (LDH) from baseline (immediately before eccentric exercise) to the end of the treatment (at 96 hours after eccentric exercise), and the muscle damage markers (serum CK and serum LDH activity) were measured immediately before (baseline), and at 24, 48, 72, and 96 hours after eccentric exercise.

**Figure 1:**
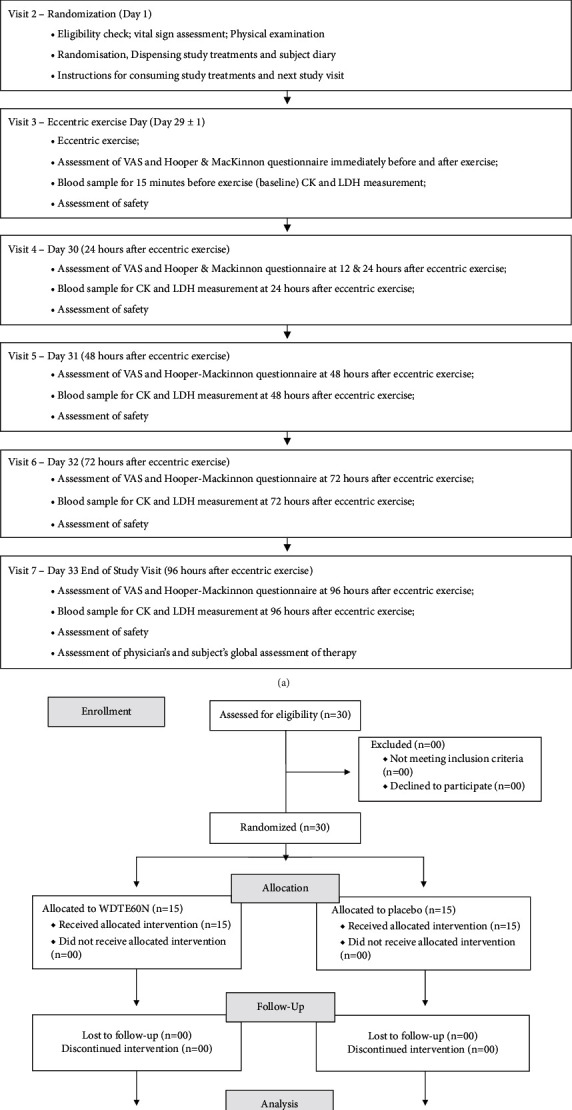
(a) Flow of the study and (b) subject disposition.

The subject's and physician's global assessment of therapy was assessed at the end of treatment (at 96 hours after eccentric exercise) (Figure 1(a)).

The safety assessment of the study products was based on the adverse events reported by changes in vital parameters, laboratory investigations (complete blood count, liver function test, and renal function test), and the general well-being of subjects.

### 2.5. Statistical Analysis

This was an exploratory study and the sample size was calculated based on the published literature [22]. Considering the power of 80%, alpha of 0.05, and anticipated mean VAS score for the WDTE60N group and placebo group of 2.88 and 3.36, respectively, a sample size of 28 subjects was required and after including 10% of dropouts, the total sample size was estimated to be 30 subjects.

Subjects were included in the per-protocol population if they completed their study treatment without any major protocol deviations and had treatment compliance of at least ≥80%. The results were presented as mean ± standard deviation for continuous variables and counts and percentages for categorical variables. Kolmogorov–Smirnov test was performed to assess the normality distribution of the data. The unpaired *t*-test was used for intra-treatment group comparison depending on the normality distribution of continuous. The study involves efficacy evaluation of the test product in DOMS, a condition in which the pathogenesis is known to start-attain peak-and decline over a period of time after performing an unaccustomed eccentric muscular activity, hence the assessment of product efficacy cannot be significantly established at any single time point after eccentric exercise. Therefore, the area under the curve (AUC) was utilized to describe the span of the efficacy parameters over time after the DOMS induction. A *p* value of <0.05 was considered statistically significant. Statistical analysis was performed using SAS version 9.4 (SAS Institute Inc., USA).

## 3. Results

From 29 Dec 2021 to 06 Jan 2022, 30 recreationally active subjects (mean age: 28.23 ± 4.20 years; males: 21; females: 9) were screened, enrolled, and randomly allocated to either the WDTE60N group (*n* = 15) or the placebo group (*n* = 15). All subjects completed the study (Figure 1(b)). Demographic and baseline clinical characteristics of the study population are given in Table 1.

Both groups had statistically insignificant and comparable for efficacy parameters (VAS score, all domains of well-being status, serum CK, and LDH concentration) when evaluated immediately before exercise.

### 3.1. Visual Analog Scale

**Figure 2:**
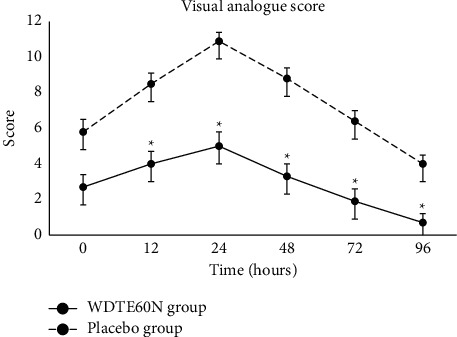
Changes in VAS score immediately after (0 hour) and 12–96 hours (12, 24, 48, 72, and 96 hours) after eccentric exercise in the subjects receiving WDTE60N and placebo; values are presented as mean ± SD (*n* = 15 for each group); ^*∗*^*p* < 0.05.

Both treatment groups had statistically insignificant and comparable VAS scores immediately after eccentric exercise (baseline). The VAS score was significantly lowered in the WDTE60N group compared to the placebo group at 12 hours (4.0 ± 0.7 vs. 4.5 ± 0.6; *p*=0.0320), 24 hours (5.0 ± 0.8 vs. 5.9 ± 0.5; *p*=0.0010), 48 hours (3.3 ± 0.7 vs. 5.5 ± 0.6; *p* < 0.0001), 72 hours (1.9 ± 0.7 vs. 4.5 ± 0.6; *p* < 0.0001) and 96 hours (0.7 ± 0.5 vs. 3.3 ± 0.5, *p* < 0.0001) after exercise (Figure 2). Mean AUC from immediately after exercise to the end of the study (AUC_0–96h_) was significantly lower in the WDTE60N group compared to the placebo group (286.8 ± 46.7 vs. 460 ± 40.5; *p* < 0.0001) (Supplementary Figure 1).

### 3.2. Assessment of Well-Being Status Using an Adapted Version of the Hooper and MacKinnon Questionnaire

**Figure 3:**
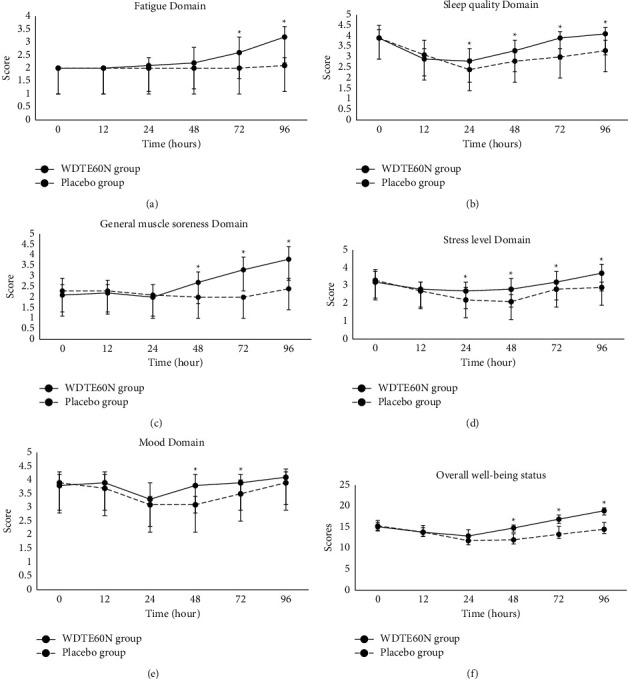
Changes in the well-being status assessed using an adapted version of the Hooper and MacKinnon questionnaire immediately after (0 hour) and 12–96 hours (12, 24, 48, 72, and 96 hours) after eccentric exercise in subjects who received WDTE60N and placebo; values are presented as mean ± SD (*n* = 15 for each group); ^*∗*^*p* < 0.05. (a) Changes in the fatigue domain, (b) sleep quality domain, (c) general muscle soreness, and (d) stress level domain immediately after (0 h) and 12–96 hours (12, 24, 48, 72, and 96 hours) after eccentric exercise in subjects who received WDTE60N and placebo; values are presented as mean ± SD (*n* = 15 for each group); ^*∗*^*p* < 0.05. (e) Changes in mood domain immediately before, immediately after (0 hour), and 12–96 hours (12, 24, 48, 72, and 96 hours) after eccentric exercise in subjects who received WDTE60N and placebo; values are presented as mean ± SD (*n* = 15 for each group); ^*∗*^*p* < 0.05. (f) Changes in the overall well-being status immediately after (0 hour) and 12–96 hours (12, 24, 48, 72, and 96 hours) after eccentric exercise in the subjects receiving WDTE60N and placebo; values are presented as mean ± SD (*n* = 15 for each group); ^*∗*^*p* < 0.05.

Both treatment groups were comparable immediately before and after eccentric exercise, as was evident by no statistically significant difference seen for all five domains and overall well-being at baseline (*p* > 0.05) (Figure 3). However, there was a significant improvement in all five domains at different time points as follows:

### 3.3. Fatigue

The fatigue score was significantly higher at 72 hours (2.6 ± 0.6 vs. 2.0 ± 0; *p*=0.0025) and 96 hours (3.2 ± 0.4 vs. 2.1 ± 0.3; *p* < 0.0001) after the exercise in the WDTE60N group compared to the placebo group.

### 3.4. Sleep Quality

The WDTE60N group reported significantly higher scores at 24 hours (2.8 ± 0.6 vs. 2.4 ± 0.5; *p*=0.0499), 48 hours (3.3 ± 0.5 vs. 2.8 ± 0.4; *p*=0.0032), 72 hours (3.9 ± 0.3 vs. 3.0 ± 0.4; *p* < 0.0001) and 96 hours (4.1 ± 0.3 vs. 3.3 ± 0.5; *p* < 0.0001) after exercise.

### 3.5. General Muscle Soreness

There was a significantly higher general muscle soreness score at 48 hours (2.7 ± 0.5 vs. 2.0 ± 0.0; *p*=0.0001), 72 hours (3.3 ± 0.6 vs. 2.0 ± 0.0; *p* < 0.0001), and 96 hours (3.8 ± 0.6 vs. 2.4 ± 0.5; *p* < 0.0001) after exercise in the WDTE60N group compared to the placebo group.

### 3.6. Stress Levels

The scores for stress levels were significantly higher in the WDTE60N group compared to the placebo group at 24 hours (2.7 ± 0.5 vs. 2.2 ± 0.7; *p*=0.0388), 48 hours (2.8 ± 0.6 vs. 2.1 ± 0.4; *p*=0.0005), 72 hours (3.2 ± 0.6 vs. 2.8 ± 0.4; *p*=0.0345), and 96 hours (3.7 ± 0.5 vs. 2.9 ± 0.3; *p* < 0.0001) after exercise.

### 3.7. Mood Levels

Compared to the placebo group, the WDTE60N group showed significantly higher mood scores at 48 hours (3.8 ± 0.4 vs. 3.1 ± 0.3; *p* < 0.0001), and 72 hours (3.9 ± 0.3 vs. 3.5 ± 0.5; *p*=0.0051) after exercise.

### 3.8. Overall Well-Being

The overall well-being scores were significantly higher at 48 hours (14.8 ± 1.4 vs. 12.0 ± 0.7; *p* < 0.0001) and 72 hours (16.9 ± 1.9 vs. 13.3 ± 1.0; *p* < 0.0001) and 96 hours (18.9 ± 1.6 vs. 14.5 ± 0.7; *p* < 0.0001) after exercise.

Corresponding mean AUC for the aforementioned domains was also significantly higher in the WDTE60N group compared to the placebo group (Supplementary Figure 2).

### 3.9. Serum Creatine Kinase Concentration

There was no statistically significant difference in the preexercise serum CK concentration between the WDTE60N group and the placebo group (115.3 ± 26.5 IU/L and 170.0 ± 136.6 IU/L; *p*=0.1483). Serum CK concentrations peaked at 48 hours (152.9 ± 52.4 IU/L) but decreased at 72 hours (125.9 ± 41.2 IU/L) and 96 hours (138 ± 53.6 IU/L) after eccentric exercise in the WDTE60N group. Contrastingly, in the placebo group, there was a decrease in serum CK concentration initially at 24 hours and 48 hours followed by an increase at 72 hours and 96 hours after exercise. A decrease in the mean AUC_0–96h_ serum CK was observed in the WDTE60N group compared to the placebo group. However, the difference between both treatment groups at different time points and in AUCs was statistically insignificant.

### 3.10. Serum LDH Concentrations

**Figure 4:**
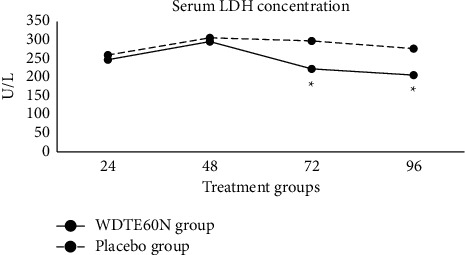
Changes in serum LDH activity after 24–96 hours (24, 48, 72, and 96 hours) of eccentric exercise in the subjects receiving WDTE60N and placebo; values are presented as mean ± SD (*n* = 15 subjects in the WDTE60N group; *n* = 14 subjects in the placebo group); ^*∗*^*p* < 0.05.

Preexercise serum LDH concentration was 262.3 ± 151.3 U/L and 253.8 ± 72.0 U/L for the WDTE60N and placebo groups, respectively, and the difference was statistically insignificant. Serum LDH concentration peaked at 48 hours and decreased gradually at 72 hours and 96 hours after exercise in both groups (Figure 4). It significantly lowered in subjects in the WDTE60N group at 72 hours (223.2 ± 38.5 vs. 298.5 ± 95.0; *p*=0.0106) and 96 hours (206.6 ± 25.4 U/L vs. 277.8 ± 98.6 U/L; *p*=0.0156) compared to those in the placebo group. A statistically significant decrease in the mean AUC_0–96h_ serum LDH concentration was observed in the WDTE60N group compared to the placebo group (23623.7 ± 2532.0 vs. 26138.6 ± 3669.5; *p*=0.0446) (Supplementary Figure 3).

### 3.11. Determination of the Subject's and Physician's Global Assessment of Therapy

The subject's global assessment of therapy in both WDTE60N and placebo groups was 4.00 ± 0.0 and 2.00 ± 0.0, respectively (*p* < 0.05). Similarly, the physician's global assessment of therapy showed significantly higher scores for the WDTE60N group compared to the placebo group (4.07 ± 0.26 vs. 2.40 ± 0.51; *p* < 0.0001).

### 3.12. Safety and Tolerability of the Study Treatments

No incidence of serious adverse events was reported during the entire study, and the study treatments were well tolerated by the subjects. There were no clinically significant changes in vital signs, routine examinations of hematology, liver function test, and renal function test in the study subjects of both treatment arms.

## 4. Discussion

The study outcomes demonstrated the effects of the WDTE60N on muscle soreness and muscle damage markers after intensive eccentric exercise in recreationally active subjects compared to placebo. The main findings of our study included the following: significantly low pain intensity after eccentric exercise among the subjects receiving WDTE60N compared to those receiving placebo at 12, 24, 48, and 96 hours after eccentric exercise; significantly higher improvement in fatigue, mood, sleep quality, generalized muscle soreness, stress, and overall well-being (assessed by the adapted Hooper and MacKinnon questionnaire) in subjects receiving WDTE60N than placebo; significantly decreased serum LDH concentrations but not serum CK concentration with consistent WDTE60N supplementation before and after eccentric exercise; and significantly higher ratings for WDTE60N in the subject's and physician's global assessment of therapy. Furthermore, the study treatments were well tolerated by all subjects, and no serious adverse events were reported during the study period.

Recently, phytotherapy has received attention in medicine globally. Considering their tremendous therapeutic benefits and a wide margin of safety, herbal products have been increasingly used by different diseased and healthy populations [23–26]. Several studies demonstrated the efficacy of curcumin in reducing muscle soreness or pain associated with exercise-induced muscle damage [27–30]. The comparison of acute pain-relieving effects of curcumin (400 mg), nimesulide, and acetaminophen showed comparable analgesic activity of curcumin with that of acetaminophen. Furthermore, curcumin also possesses better gastric tolerability than nimesulide [31]. In this study, assessment of pain intensity using the VAS score demonstrated significantly lower pain in subjects receiving WDTE60N throughout the postexercise period compared to those receiving placebo. Notably, this was evident (*p* < 0.05) within 12 hours of eccentric exercise and indicated faster recovery. Similarly, an assessment of the well-being status using the adapted version of the Hooper and MacKinnon questionnaire confirmed less muscle soreness in subjects receiving WDTE60N (*p* < 0.05). Moreover, overall well-being and other aspects such as fatigue, mood, sleep quality, and stress were also significantly improved after exercise in subjects, who received WDTE60N than those, who were administered a placebo. The impact of WDTE60N on stress levels was as per a previous study. Sciberras et al. reported a higher number of subjects with “better than usual” scores in the subjective assessment of psychological stress during the training day when subjects received curcumin supplementation before training [32]. Furthermore, those subjects, who feel the pain and the physicians treating it may have different assessments and perceptions of the pain, which could affect the evaluation of treatment efficacy. Thus, this needs to be evaluated from both the subject's and the physician's perspectives. In this study, both the subject's and the physician's ratings for global assessment of therapy were significantly higher for WDTE60N. This confirms that both subjects and physicians perceived WDTE60N as an effective treatment option to counter the physiological effects of DOMS. The pain-alleviating efficacy of WDTE60N was also demonstrated in the management of chronic knee pain. The study also demonstrated improvements in joint performance and mobility without any significant safety concerns following WDTE60N administration once daily for 3 months [17]. Thus, the results of previous and current studies reflect that WDTE60N can have beneficial effects on the entire musculoskeletal system with a single daily dose of one capsule.

Serum CK concentration increased after eccentric exercise as it is well distributed in the muscle tissue and is released into the circulation according to the loss of sarcolemmal integrity as a result of the mechanical stress of eccentric exercise or metabolic causes (glycogen depletion). Similarly, increased serum LDH concentrations indicate cell damage as LDH present in the cytoplasm catalyzes the reversible process of pyruvate to lactate under anaerobic conditions. Thus, an increase in serum CK or LDH activities are indirect markers for muscle damage. In this study, serum CK and LDH concentrations increased and attained the highest levels at 48 hours after exercise in both treatment groups indicating that DOMS was a clinical condition with peak clinical implications within 72 hours of eccentric exercise. Serum LDH concentration significantly lowered in the WDTE60N group compared to the placebo group at 72 and 96 hours after exercise. Similarly, serum CK activity was also reduced in subjects, who received WDTE60N compared to placebo (*p* > 0.05). Results from several studies have warranted the need to evaluate the effect of curcumin supplements on muscle damage markers to determine the correlation between frequent/higher dosing with a decrease in muscle damage markers. A study evaluated the effects of a curcumin and piperine combination on the recovery kinetics after exercise-induced muscle damage and reported no effect on serum CK concentration [33]. Similarly, Drobnic et al. determined the effects of curcumin (initiated 48 hours before exercise and continued for 24 hours after exercise) compared with placebo on exercise-induced DOMS and reported no difference in CK concentration between both groups but noted significantly lowered levels of interleukin-8 in those, who were given curcumin after exercise [34].

While evaluating the safety, the present study reveals that WDTE60N intake for 33 days neither caused any adverse effects nor reported any change in vital/laboratory parameters. These observations confirm that WDTE60N is safe and tolerable for human consumption. However, nonsteroidal anti-inflammatory drugs, a commonly used treatment strategy to relieve muscle soreness associated with DOMS by athletes, has several side effects.

Briefly, intense muscle soreness decreases strength and muscle power that ultimately pushing the athletes to perform the next bout of exercise with higher intensity than normally habituated [35]. This increased intensity due to damaged and weakened muscle fibers (alterations in muscle sequencing and recruitment patterns) after eccentric exercise will likely increase the risk for further injury if a premature return to exercise is attempted [36]. Based on the results of the present study, we believe that WDTE60N intake is advantageous to athletes as it decreases subjective and objective parameters of muscle soreness. Furthermore, it also reduces stress levels and improves the performance of athletes.

### 4.1. Limitations

We acknowledge the limitations of this study, as the present study did not determine the effect of therapy on athletes, who regularly train and compete. Future studies can be planned to evaluate the efficacy of the WDTE60N in such athletes.

## 5. Conclusion

This study demonstrated that administering WDTE60N both before and after eccentric exercise reduces postexercise pain, improves subject well-being (including muscle soreness) and decreases lactate accumulation, which is demonstrated by significantly lowered serum LDH activity without any major safety concern in recreationally active subjects. However, the effect of WDTE60N administration on serum CK levels needs to be investigated in a larger population.

## Figures and Tables

**Table 1:** The demographics and baseline clinical characteristics of the study population (*n* = 30 subjects).

Characteristics	Overall (*N* = 30)	WDTE60N group (*n* = 15)	Placebo group (*n* = 15)
Age (years), mean ± SD	28.23 ± 4.20	29.7 ± 3.6	26.8 ± 4.3
Number of male subjects	21	11	10
Number of female subjects	9	4	5
Weight (kg), mean ± SD	59.98 ± 3.21	60.27 ± 3.58	59.69 ± 2.89
Height (cm), mean ± SD	170.84 ± 6.37	170.73 ± 6.37	170.95 ± 6.58
Body mass index (kg/m^2^), mean ± SD	20.59 ± 1.16	20.72 ± 1.28	20.45 ± 1.04

## Data Availability

The data generated or analyzed during this study are included within the article and supplementary file.

## Supplementary Materials

Supplementary Materials Supplementary Table 1. CONSORT 2010 checklist of information to include when reporting a randomized trial. Supplementary Figure 1. Mean area under the curve (AUC) for 0–96 hours of VAS score; values are presented as mean ± SD (*n* = 15 for each group). Supplementary Figure 2. Assessment of well-being using adapted the Hooper and MacKinnon questionnaire; immediately, postworkout timepoint is considered as 0 hour. Supplementary Figure 2 (A). Mean area under the curve (AUC) for 0–96 hours of the score for fatigue domain as assessed by the Hooper and MacKinnon questionnaire; values are presented as mean ± SD (*n* = 15 for each group). Supplementary Figure 2 (B). Mean area under the curve (AUC) for 0–96 hours of the score for sleep quality domain as assessed by the Hooper and MacKinnon questionnaire; values are presented as mean ± SD (*n* = 15 for each group). Supplementary Figure 2 (C). Mean area under the curve (AUC) for 0–96 hours of the score for general muscle soreness domain as assessed by the Hooper and MacKinnon questionnaire; values are presented as mean ± SD (*n* = 15 for each group). Supplementary Figure 2 (D). Mean area under the curve (AUC) for 0–96 hours of score for stress domain as assessed by the Hooper and MacKinnon questionnaire; values are presented as mean ± SD (*n* = 15 for each group). Supplementary Figure 2 (E): Mean area under the curve (AUC) for 0–96 hours of the score for mood domain as assessed by the Hooper and MacKinnon questionnaire; values are presented as mean ± SD (*n* = 15 for each group). Supplementary Figure 2 (F). Mean area under the curve (AUC) for 0–96 hours of the score for overall well-being status score as assessed by the Hooper and MacKinnon questionnaire; values are presented as mean ± SD (*n* = 15 for each group). Supplementary Figure 3. Mean area under the curve (AUC) for 24–96 hours of serum LDH concentration; values are presented as mean ± SD (*n* = 14 for each group; one subject from each group was excluded from the analysis due to anomalous observation at baseline and at 48 hours after eccentric exercise).
