# Supplementary material for: Efficacy and Safety of TurmXTRA® 60N in Delayed Onset Muscle Soreness in Healthy, Recreationally Active Subjects: A Randomized, Double-Blind, Placebo-Controlled Trial
Source: Evid Based Complement Alternat Med. 2022 Aug 5;2022:9110414. doi: 10.1155/2022/9110414 (PMC9374544; doi:10.1155/2022/9110414)
Supplement: Supplementary Materials — Supplementary Table 1. CONSORT 2010 checklist of information to include when reporting a randomized trial. Supplementary Figure 1. Mean area under the curve (AUC) for 0–96 hours of VAS score; values are presented as mean ± SD (n = 15 for each group). Supplementary Figure 2. Assessment of well-being using adapted the Hooper and MacKinnon questionnaire; immediately, postworkout timepoint is considered as 0 hour. Supplementary Figure 2 (A). Mean area under the curve (AUC) for 0–96 hours of the score for fatigue domain as assessed by the Hooper and MacKinnon questionnaire; values are presented as mean ± SD (n = 15 for each group). Supplementary Figure 2 (B). Mean area under the curve (AUC) for 0–96 hours of the score for sleep quality domain as assessed by the Hooper and MacKinnon questionnaire; values are presented as mean ± SD (n = 15 for each group). Supplementary Figure 2 (C). Mean area under the curve (AUC) for 0–96 hours of the score for general muscle soreness domain as assessed by the Hooper and MacKinnon questionnaire; values are presented as mean ± SD (n = 15 for each group). Supplementary Figure 2 (D). Mean area under the curve (AUC) for 0–96 hours of score for stress domain as assessed by the Hooper and MacKinnon questionnaire; values are presented as mean ± SD (n = 15 for each group). Supplementary Figure 2 (E): Mean area under the curve (AUC) for 0–96 hours of the score for mood domain as assessed by the Hooper and MacKinnon questionnaire; values are presented as mean ± SD (n = 15 for each group). Supplementary Figure 2 (F). Mean area under the curve (AUC) for 0–96 hours of the score for overall well-being status score as assessed by the Hooper and MacKinnon questionnaire; values are presented as mean ± SD (n = 15 for each group). Supplementary Figure 3. Mean area under the curve (AUC) for 24–96 hours of serum LDH concentration; values are presented as mean ± SD (n = 14 for each group; one subject from each group was excluded from the analysis due to anoma [file 9110414.f1.zip › Supplementary Figure 2.docx]

**Supplementary Figure 2:** Assessment of wellbeing using adapted Hooper & MacKinnon Questionnaire; Immediately post workout timepoint is considered as 0 hour

**Supplementary Figure 2 (A):** Mean area under the curve (AUC) for 0–96 hours of score for fatigue domain as assessed by Hooper & MacKinnon Questionnaire; Values are presented as mean±SD (n=15 for each group)

**Supplementary Figure 2** **(B):** Mean area under the curve (AUC) for 0–96 hours of score for sleep quality domain as assessed by Hooper & MacKinnon Questionnaire; Values are presented as mean±SD (n=15 for each group)

**Supplementary Figure 2** **(C):** Mean area under the curve (AUC) for 0–96 hours of score for general muscle soreness domain as assessed by Hooper & MacKinnon Questionnaire; Values are presented as mean±SD (n=15 for each group)

**Supplementary Figure 2** **(D):** Mean area under the curve (AUC) for 0–96 hours of score for stress level domain as assessed by Hooper & MacKinnon Questionnaire; Values are presented as mean±SD (n=15 for each group)

**Supplementary Figure 2** **(E):** Mean area under the curve (AUC) for 0–96 hours of score for mood domain as assessed by Hooper & MacKinnon Questionnaire; Values are presented as mean±SD (n=15 for each group)

**Supplementary Figure 2** **(F):** Mean area under the curve (AUC) for 0–96 hours of score for overall wellbeing status score as assessed by Hooper & MacKinnon Questionnaire; Values are presented as mean±SD (n=15 for each group)
